# A Concept Using α‐Niche Evolution Within Bacterial Communities to Direct β‐Niche Evolution of Focal Species

**DOI:** 10.1111/1462-2920.70255

**Published:** 2026-02-25

**Authors:** Thomas Scheuerl, Damian W. Rivett

**Affiliations:** ^1^ Research Department for Limnology University of Innsbruck Mondsee Austria; ^2^ Department of Natural Sciences, Faculty of Science and Engineering Manchester Metropolitan University Manchester UK

## Abstract

The process of bacterial adaptation has a profound impact on human wellbeing and health, but our toolkit to modify evolution is limited. Here, we present a concept of how steering adaptation can be achieved by integration of bacterial evolution and microbial ecology. The fundamental question is how specific species bloom after community perturbation and subsequently evolve. We consider two kinds of traits—α‐niche traits involved in partitioning resources (e.g., broadened resource consumption) and β‐niche traits driven by changes in the abiotic environment (e.g., pH adaptation or resistance after antibiotic treatment). We suggest that the evolution of the second trait can be directed indirectly via the evolution of the first trait, exploiting specific interspecies interactions. Thus, understanding how these traits interact in co‐evolving communities may offer unprecedented opportunities to deflect trait evolution. Summarising current knowledge, emphasising open questions and highlighting conceptual ideas, we hope to stimulate new studies that are needed to move this field forward.

## Graphical abstract

There is a great need for approaches to control how species evolve in response to environmental changes, particularly in complex microbiomes. The feasibility is often met with scepticism due to the multitude of open questions and high dimensionality of community eco‐evolutionary dynamics. Attempts to direct evolution in microbiomes may seem elusive, but our synthesis of the field delivers promising ways to overcome these problems. We start our work by summarising how microbiomes impact evolutionary change over ecological timescales before building a concept that rationalises ecological and evolutionary approaches. We state how this combined approach will generate new and exciting knowledge that will have a multifaceted impact from healthcare to environmental bioremediation. The concept describes how evolution can be directed by biotic interactions that modify ecological opportunities. We detail how and when evolution to exploit a novel resource, the evolution of α‐niches, mediates the evolution of a trait needed to mitigate abiotic environmental change, the evolution of β‐niches, and how this plays out in the context of a community. A worked example visualises how applying the concept can create opportunities that minimise the evolution of antibiotic resistance, and another example visualises how this could be exploited for bioremediation. We then summarise how biotic interactions impact evolutionary rates more generally before proposing the focus of future research; this highlights new avenues to illuminate the ‘black box’ of the ecological niches of microbes, and suggests experimental designs that shift the eco‐evolutionary standard.

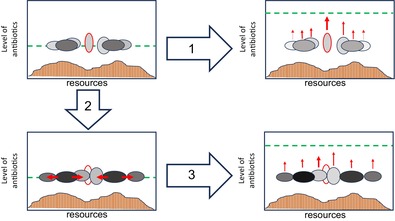

## 1 Microbial Eco‐Evolution Has a Value

In recent years, a multitude of research has shown that ecological forces shape the composition and functionality of bacterial communities in predictable ways (Catalán et al. 2021; Estrela, Vila, et al. 2021; Gralka et al. 2020; Pascual‐García et al. 2025; Thompson et al. 2017). This realisation has led health‐care systems, public sectors and politicians to acknowledge the importance of a whole community perspective (Cavicchioli et al. 2019). For instance, a recently published call across a range of journals suggests that microbial communities should be deployed against the climate catastrophe because of their huge physiological and adaptive potential (Peixoto et al. 2024). Similarly, the ecological benefits a healthy gut microbiome provides are now widely appreciated, and interventions building on complex communities are being developed to improve gut functioning (de la Cuesta‐Zuluaga et al. 2024; Maier et al. 2021). Understanding how evolution plays out in natural bacterial communities is important, as bacterial lineages also evolve over ecological timescales (Good and Rosenfeld 2023; Zhao et al. 2019). For example, bacterial evolution has direct impacts on human health through antibiotic resistance and the evolution of virulence in long‐term patients (Smith et al. 2006; Wheatley et al. 2021). Many decades of study have uncovered the genetic mechanisms of evolution in monoculture experiments (Barrick et al. 2009; Tenaillon et al. 2016). Yet, all bacteria live in diverse communities (Box 1), and multiple interspecies interactions within microbiomes can impact evolutionary processes in different ways (Bailey et al. 2013; Lawrence et al. 2012). Exploiting approaches that consider eco‐evolutionary interactions in communities may be vital to modify evolutionary changes observed in nature (Barraclough 2015). However, due to the complexity and high‐dimensionality of community eco‐evolutionary dynamics and the multitude of open questions, research combining ecological and evolutionary processes is often met with great scepticism (Crocker et al. 2023). Here, we outline approaches that can address some of these concerns and promote knowledge to help manipulate bacterial communities with improved functions.

## 2 How Communities Impact Bacterial Species Evolution

With in vitro models, we can follow the process of bacterial adaptation in real time and watch evolution in action (Good et al. 2017; Rainey and Travisano 1998). Bacterial isolates rapidly adapt to new conditions, and fitness continues to increase even over several thousand generations (Tenaillon et al. 2016; Wiser et al. 2013). While the effect size of beneficial mutations seems to reduce over long time scales (Couce et al. 2024), there can still be a regular inflow of beneficial mutations (Barrick et al. 2009). However, unlike most in vitro experimental approaches, in situ bacteria rarely evolve in isolation but co‐evolve with many other organisms within the same habitat (Chase et al. 2021; Rohwer et al. 2025). Even when bacterial communities are strongly disrupted by chemical addition or immigration, deterministic and stochastic processes combine to allow different species to survive, binding them in a complex co‐evolving network during community re‐assembly (Ravi et al. 2019). Particularly after disruption, early immigrants may monopolise open niches (De Meester et al. 2002), which is speculated to drive evolutionary changes (Zhao et al. 2019). These dynamics of evolutionary changes in natural environments have been revealed by in situ lineage tracking by molecular approaches (Bendall et al. 2016; Rohwer et al. 2025), and even in our gut microbiome, we can see similar dynamics (Madi et al. 2023; Wheatley et al. 2021; Zhao et al. 2019). While the influence of the background community on the evolution of individual bacterial species is now accepted, the precise mechanisms behind this are still puzzling.

### 2.1 The Role of Biodiversity

**FIGURE 1:**
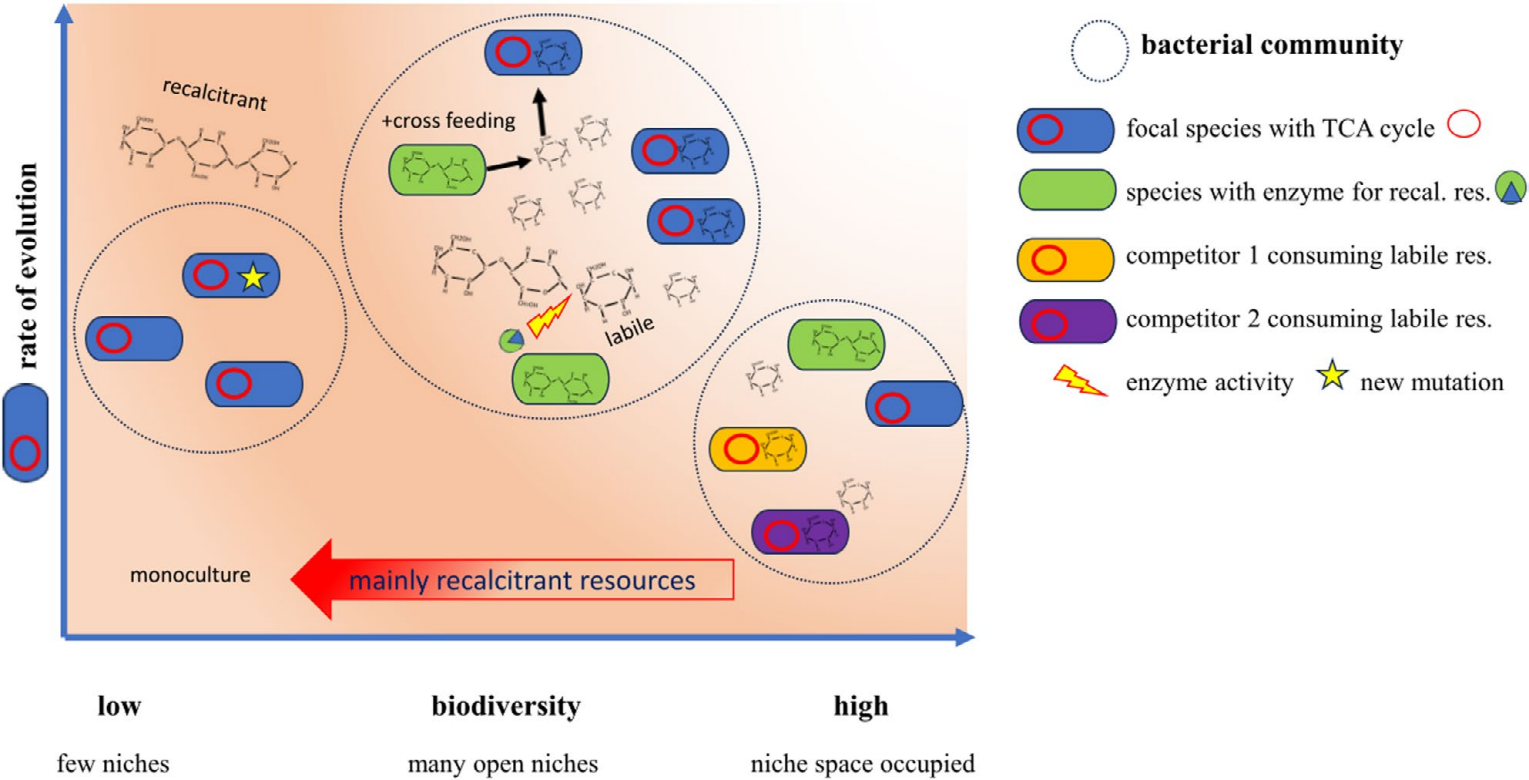
Biodiversity can beget and constrain evolution. The adaptive rate of a species depends on the interaction with the surrounding community. A focal species (blue species) that is isolated (e.g., monoculture) may experience few limitations to evolve, but in nature, the current environments can be unfavourable (e.g., full of recalcitrant resources). To unfold initial evolutionary potential, existing traits (e.g., TCA cycle, red circle) may need refinement to meet the current situation. Otherwise, adaptation may depend on the evolution of completely new ‘key‐innovations’ (yellow star) that do not yet exist (break down of recalcitrant resource). When biodiversity increases, it is more likely that some species (e.g., green species) occur that act as ‘ecosystem engineers’ and are able to break down difficult material by specialised exoenzymes (green–blue dot), or produce new niches via metabolic overflow (Bajic and Sanchez 2020), which results in ‘cross‐feeding’. This process creates more niches, which are potentially simpler to exploit (e.g., more labile resources), which facilitates refinement of the existing trait (red circles). While this process of creating more niches probably continues with increasing biodiversity, it also becomes more likely that other species (purple and orange species) consume and fill these niches and thus again constrain ecological opportunities. So far, data support such a *humped‐shaped relationship*, with the result that at low levels of biodiversity, evolution is facilitated, but this plateaus at very high levels of diversity.

The diversity of a community seems to play an important role in mediating the availability of resources, and it looks like in low‐diversity communities, evolution is facilitated, for example, by niche construction, whereas in highly diverse communities, niche filling may limit ecological opportunities (Figure 1). Supporting this, one study found a plateauing increase of taxonomic biodiversity in communities with increased taxonomic diversity (Madi et al. 2020), and follow‐up work suggested that more genetic variants of a species are found in human gut microbiomes with reduced biodiversity (Madi et al. 2023). At what level of biodiversity does there come a switch from facilitation to inhibition of evolution? This is a pertinent, yet unexplored question. In general, taxonomic diversity seems not to be the only determinant of directing adaptive rates (Scheuerl et al. 2020). From a theoretical perspective, the survival of new mutations is dependent on the size of the community and the saturation of niches (McEnany and Good 2024), and another model suggested that changes in the evolution rate depend on the shape of trade‐offs in resource use (Barraclough 2019a).

### 2.2 Creating and Consuming Niches

The mechanistic question of whether biodiversity constrains or begets biodiversity is a fundamental one. The contradiction is often explained by how biodiversity manipulates niches (Schluter and Pennell 2017). Likewise, whether bacteria evolve more in diverse communities (Jousset et al. 2016; Lawrence et al. 2012) or whether adaptive rates are constrained in communities (Gómez et al. 2016; Klümper et al. 2019; Scheuerl et al. 2020) potentially hinges on the availability of niches. So, can we postulate how resources and biodiversity together shape evolution? One experimental study using four organoheterotroph bacteria, from phylogenetically different lineages, found that while species that evolved in monoculture remained competitive, facilitative interactions, based on cross‐feeding, evolved in simplified communities (Lawrence et al. 2012). Some bacterial species consumed carbon resources but excreted metabolites that other species evolved using, thus minimising resource competition. The study provided evidence that interspecific interactions can initiate the evolution of cross‐feeding via metabolite secretion, resulting in new niches that were more accessible than resources present in the medium. A comparable experiment used eight different ones.

*Pseudomonas* strains and suggested that resource competition fostered evolutionary diversification provided that sufficient alternative resources were available, therefore reducing antagonistic interactions (Jousset et al. 2016). By using different strains from the same species, however, community members competed for a narrow set of resources, and this intra‐specific competition is a likely motor to promote adaptive radiations (Rivett et al. 2017). In contrast, another study tracked the evolution of a diverse set of focal species that were embedded in natural communities and found that diversity constrained adaptation (Scheuerl et al. 2020). Here, a close interaction between the focal species and the communities was detected; species with larger genomes evolved more while more diverse communities constrained evolution. In line with this finding, a similar study reported inhibited diversification of a focal species in natural soil communities as ecological opportunities were consumed (Gómez and Buckling 2013).

### 2.3 Co‐Evolution and Interactions

With a focus on the availability of resources, one work showed that competitors can increase the adaptive radiation of *P. fluorescence* initially, but if the competitors themselves evolve and occupy niches, this again limits evolutionary potential (Bailey et al. 2013). Building on this, co‐evolving species of wheat‐straw‐cultured communities were found to limit the evolutionary potential of focal species and constrained evolution towards less rewarding resources (Evans et al. 2020). There are ongoing discussions as to whether positive (e.g., mutualistic) or negative (e.g., competition) interactions prevail in natural communities, and the matter is far from clear (Coyte and Rakoff‐Nahoum 2019; Foster and Bell 2012; Palmer and Foster 2022). In experimental systems, where interactions between bacteria are negative, reducing competition for resources seems to be an important mechanism (Lawrence et al. 2012; Morgan et al. 2020; Rivett et al. 2016). Bacteria are known to exploit and combat each other using chemical warfare, while supporting close alliances that provide benefits to them (Faust and Raes 2012; Thompson et al. 2017). This may affect the whole habitat or only congeners within close proximity (Dal Co et al. 2020; Gralka et al. 2020). With constant conflict and species sorting (Palmer and Foster 2022), open or newly created niches that are not filled by new invasions may rapidly be occupied by adaptive radiation of resident species if conditions are suitable (Rainey and Travisano 1998). On the other hand, there are several examples that mutualistic interactions facilitate co‐evolutionary trajectories (Gould et al. 2018; Li et al. 2021), so the sign of interactions may have an important impact on evolutionary trajectories (Box 1).

BOX 1 **Glossary.**

**Table:** 

Ecological opportunity	Ecological niches that can be occupied by a species.
α‐niches	Ecological niches in the form of resources. This could be carbon sources like glucose, leucine or other molecules.
α‐niche traits	α‐niche traits permit the coexistence between species, for example, by partitioning resource use due to different metabolic pathways.
β‐niches	These are often abiotic conditions like pH or temperature.
β‐niche traits	β‐niche traits determine survival in a particular environment.
Adaptive peaks	Adaptations lead to higher fitness of organisms. While initially rapid, the increases often slow down, which leads to marginal further increases.
Selection gradient	The vector that points to the steepest uphill direction on the adaptive landscape.
Biotic interactions	The effect that species can have on each other in a community. In our context, these interactions are often food‐mediated and can either increase or decrease the population density of partners.
Bacterial communities	Groups of bacteria that consist of many different species that have some form of interaction.
Pan‐Resource profile	A concept borrowed from the pan‐genomics of bacterial species. Resources are a communal good, and some resources, *core resources*, can be used by multiple members of a bacterial community (e.g., labile sugars or acids). For these resources, there is usually high competition. Other carbon molecules are more difficult to metabolise and require specialised metabolic pathways. Only a few species will dwell on these *accessory* substrates.

### 2.4 Biotic and Abiotic Environments

Taken together, these highlighted studies suggest an impact of biotic interactions, but they all explore how bacterial species evolve using other resources in the presence of competitors. Much less is known about how bacteria evolve to new abiotic environments, like pH or externally supplied antibiotics, in the presence of other species. There are many studies that show antibiotic resistance evolution in nature (Baquero et al. 2008; Harris et al. 2010; Karkman et al. 2019; Larsson and Flach 2022), but few explore the underlying mechanisms. One evolutionary study did show that selection of antimicrobial resistance is reduced in communities because competition adds extra physiological costs and, in parallel, also limits resource availability (Klümper et al. 2019), an aspect that was cemented by other works (Fang et al. 2023). There is also support that dual stress between antibiotic application and predation limits resistance evolution (Hiltunen et al. 2018), potentially through the competing pressures occurring when two β‐niches exert selection. Turning the perspective, another work found that communities were more resilient to changes when individual species were previously exposed to antibiotics and thus could evolve resistance (Cairns et al. 2025), which indicates that adaptations can reduce stress from biotic interactions. The bacterium *Pseudomonas fluorescens* readily radiates into different oxygen niches (Rainey and Travisano 1998), but the process was constrained by increasing biodiversity in the experimental vials (Brockhurst et al. 2007), an experiment that may also suggest evolutionary modifications to abiotic conditions by other species. Probably due to the complexity of the topic, this research direction has not developed much recently, but it needs careful expansion to meet many current challenges.

## 3 The Concept: Interplay Between Biotic Interactions and Abiotic Environments

**FIGURE 2:**
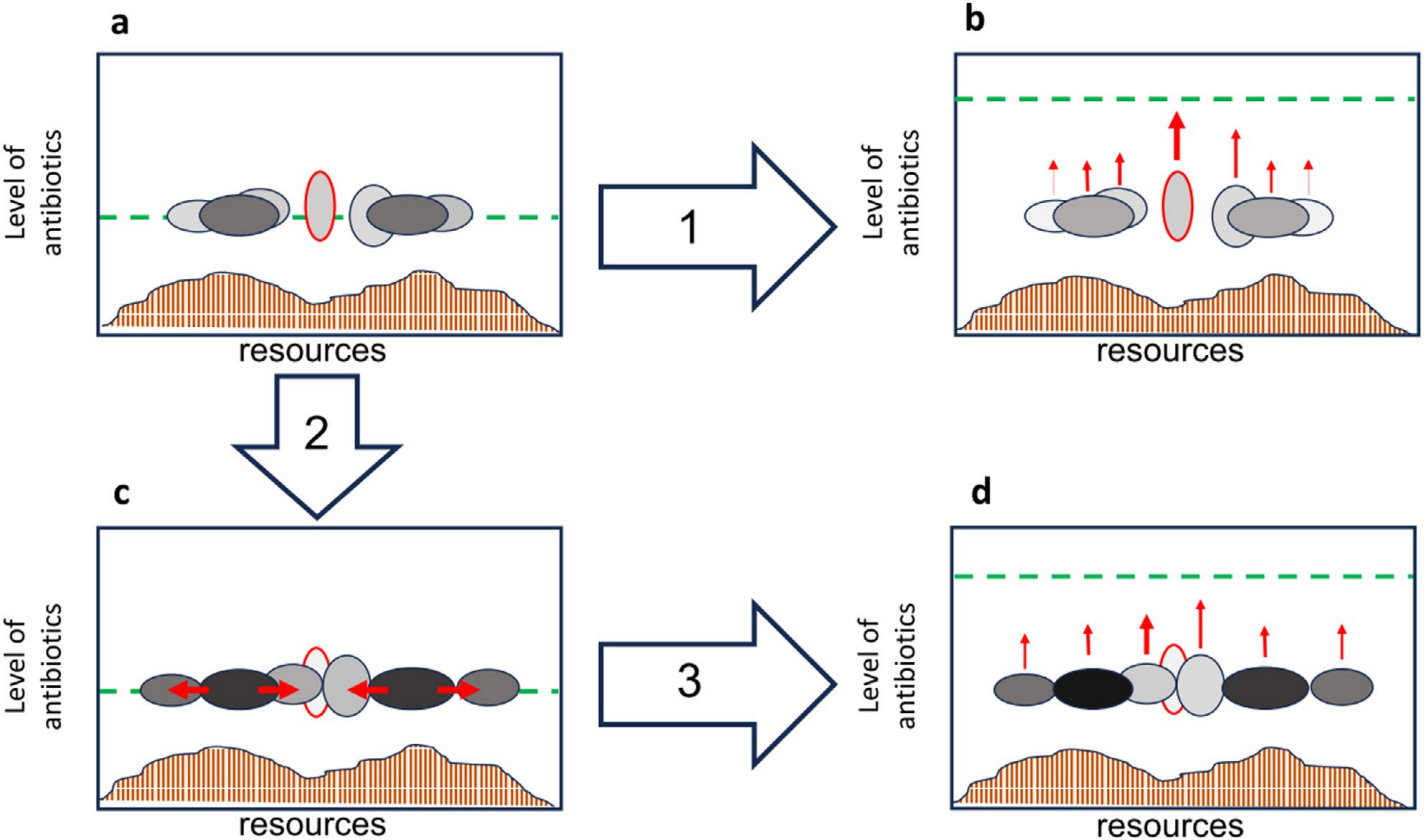
Conceptual illustration of two types of selection pressures. Starting community of seven species with their phenotype plotted on two niche axes: resources (shaded area on *X*‐axis) and antibiotics level from externally supplied treatment (green line on *Y*‐axis). The populations inherit variation in niche space (ellipsis), which indicates the evolutionary potential. More abundant species are darker; a pathogen is indicated by the red circle. (a) Species overlap in their niches but can potentially evolve to use new resources (Arrow 2) or resist increased levels of antibiotics (Arrow 1). The pathogen occupies its own peripheral niche. (b) Antibiotics increase. All species experience selection (red arrows) in the same direction (β‐niches). Co‐occurring species have little effect on antibiotic resistance evolution of the pathogen; the pathogen harbours wide genetic variation and evolves substantially (bold red arrow). Some species may have little evolutionary capacity (thin red arrows) and may go extinct. (c) Niche space evolves. Species adaptively adjust their resource spectrum (α‐niches), which leads to direct interaction between the pathogen and the co‐occurring species. For example, the light grey species experiences selection to shift to the centre because of competition with the darker species. (d) Competition limits resistance evolution. When the level of antibiotics changes under niche overlap (Arrow 3), competition may constrain resistance evolution. Now, population sizes are reduced, and interactions constrain ecological opportunities. Modified after Scheuerl et al. (2025).

Although we can measure evolutionary rates of single species when grown in tractable in vitro systems (Couce et al. 2024; Levy et al. 2015; Tenaillon et al. 2016), we not only need more data but also concepts for bacterial communities when species evolve and interact. To address this, we should first conceptualise the niche space that a heterotrophic bacterial species inhabits in terms of resources and environmental stressors. Species are located along a resource spectrum (α‐niche; Figure 2a), with differences in α‐niche traits permitting coexistence between species, for example, by partitioning resource use (Silvertown, Dodd, et al. 2006; Silvertown, McConway, et al. 2006). Some of these resources are labile and abundant; they can be easily metabolised and give a great energy yield, which often results in many species competing for these ‘pan‐resources’. Many, however, are recalcitrant and can be metabolised only with substantial effort and thus yield comparable little yield (Rivett et al. 2016), but create capacity to consume accessory niches without competition. When evolution drives trait changes around resource consumption, co‐occurring species have a direct effect on each other by limiting or facilitating ecological opportunities in the form of available substrates. Dominant species that occupy high‐yield resources and thus act as strong competitors can push other species towards alternative resources to avoid competition (Rivett and Bell 2018). On the other side, cross‐feeding and metabolite secretion may provide new ecological opportunities that species can evolve to consume (Lawrence et al. 2012). Evolution of α‐niche traits leads to niche partitioning and results in more likely coexistence between species, but also to a more exhaustive consumption of resources. Conversely, β‐niche traits determine survival in a particular environment and tend to be experienced by co‐occurring organisms in similar ways (Barraclough 2019b; Silvertown, Dodd, et al. 2006). Here, abiotic environmental factors like the temperature or pH come into play. Different species will have different tolerances for such abiotic pressures, limiting or expanding their habitat range (Chase et al. 2021; Hahn et al. 2012; Martiny et al. 2023). In such situations, a plausible prediction is that, without an overlap in α‐niches, diversity will have little impact on adaptive trait changes (Figure 2b). As such, if a species is located in its own α‐niche (no or limited resource competition), and there is no direct interference (e.g., toxic secondary metabolites), there should be little effect of species interactions on how species evolve to β‐niche changes. When, however, initially vacant α‐niches can become filled by adaptive niche trait shifts or expansions, species have to evolve to new β‐niches while α‐niches overlap with other species, which initially results in a lack of resources to grow (Figure 2c). Here, the evolution to exploit vacant resources is impacted by biodiversity, caused by various changes in the strength of selection and available genetic variation. Consequently, when competitors take ecological opportunity away in the form of available resources, this should lead to an indirect modification in the evolutionary potential of β‐niche traits, for example, by reducing the population size (Figure 2d, see Section 4). When competitors have the opportunity to co‐evolve and broaden their α‐niche trait, this should therefore impact β‐niche trait evolution in an indirect way. As a consequence, interactions over resources, like competition or cross‐feeding, can either limit or expand the ecological opportunities that a species needs to mount an adaptive response to a changing abiotic environment. When there is no option to escape the original α‐niche‐space, β‐niche traits may still evolve, but most likely at a much lower rate. In summary, we consider co‐evolution for α‐niches as a potent leverage to direct β‐niche trait evolution; thus, future studies should carefully consider how biodiversity co‐evolves in the form of resource consumption.

### 3.1 Worked Example 1: Antimicrobial Resistance

When we envision an elevation of abiotic stress, especially on microbiomes, a common, place example is during the onset of antibiotic treatment. Here, a foreign chemical is introduced to the host at high concentration, either systemically or targeted, and sensitive bacteria must respond or perish (Bell and MacLean 2018). Depending on the antibiotic, an individual bacterium may evolve resistance that renders the antibiotic ineffective in that individual but confers no resistance to others in the ecosystem (e.g., mutations in the topoisomerase/DNA gyrase preventing fluoroquinolone binding or mutations to increase efflux pump efficiency (Bhattacharyya et al. 2022)). Under ongoing selection, the modification will sweep through the population. Resistance, however, is usually associated with fitness costs (Vogwill et al. 2016), but if the sub‐population can evolve using more rewarding nutrients, within our framework, more likely in low‐diversity systems, the costs will be more easily ameliorated. The metabolic state and the availability of nutrients interact in complex ways with antibiotics (Ahmad et al. 2025). It is often observed that drugs are more efficient in actively growing populations (Bren et al. 2023), while persister cells increase in frequency under depressed metabolism (Ahmad et al. 2025). However, under healthy conditions with natural microbiomes, pathogens are commonly effectively suppressed (Wheatley et al. 2021; Zhao et al. 2019), which is likely, at least partly, due to competition between species (Spragge et al. 2023) and fitness costs of resistance (Ahmad et al. 2025). Thus, adaptation to consume resource niches, enhancing resistance evolution, is likely constrained in a community where several species are pre‐adapted to the core resource pool. With open niches potentially made vacant by species susceptible to the antibiotic, opening this pool is likely to promote the evolution of resistance. If resources are consumed by competitors, costs cannot be ameliorated easily, as metabolism was shown to constrain resistance evolution (Zampieri et al. 2017). Support for this comes from studies that found that nutrient concentration can modify success between resistant and non‐resistant bacterial strains in mixed communities (Nev et al. 2020). Moreover, many resistances are plasmid‐based, but horizontal gene transfer, particularly conjugation, which is a major source of resistance spread, is depressed under resource limitation, as the process is energy‐intensive (Lopatkin et al. 2016). Niche shifts may be particularly relevant for drugs that are imported into the cells by transporters for specific substrates (Ahmad et al. 2025).

### 3.2 Worked Example 2: Bioremediation

Outside of the medical sphere, the interplay between α‐niche and β‐niche evolution is vitally important when using microorganisms to remove pollutants. While exploiting the potential of bacterial communities has been proposed for a number of pollutants (Patel et al. 2022), the yield could be improved by embracing evolutionary and ecological processes. One example would be the removal of mercury, or heavy metals produced by acid mine drainage (Anekwe and Isa 2023), which are toxic substances leaking into environments but can be detoxified by bacterial activity. During an anaerobic process, bacteria can reduce the mercury ions into insoluble metal sulphides, which precipitate the metals and thus remove them from the environment (Nobahar et al. 2025).

In this case, supporting evolutionary processes to increase the removal of the pollutant should be enhanced by supporting the adaptive traits that exist in the community but which need refinement for higher efficiency. Biodiversity plays a crucial role so that physiological pathways for the process are active, but moreover, the community holds the potential for non‐contributing species to evolve in the desired direction. However, as previously stated (Figure 1), we would also want to avoid filling the ecosystem simply with species that contribute little to the detoxification but fill up and consume α‐niches, potentially resulting in an occupation of niches for the most productive species. Thus, if these α‐niches remain open, focal species will find an opportunity not only to thrive, but also to find space to evolve. Accordingly, a fine balance between diversity and open ecological opportunity is likely to yield the greatest effect. In such a case, we propose that an intermediate level of biodiversity will work best, as it creates open niches via cross‐feeding, houses the genetic potential for the completion of the job, and also pushes the wanted process into wanted directions.

## 4 How Interspecific Interactions Impact Evolution

**FIGURE 3:**
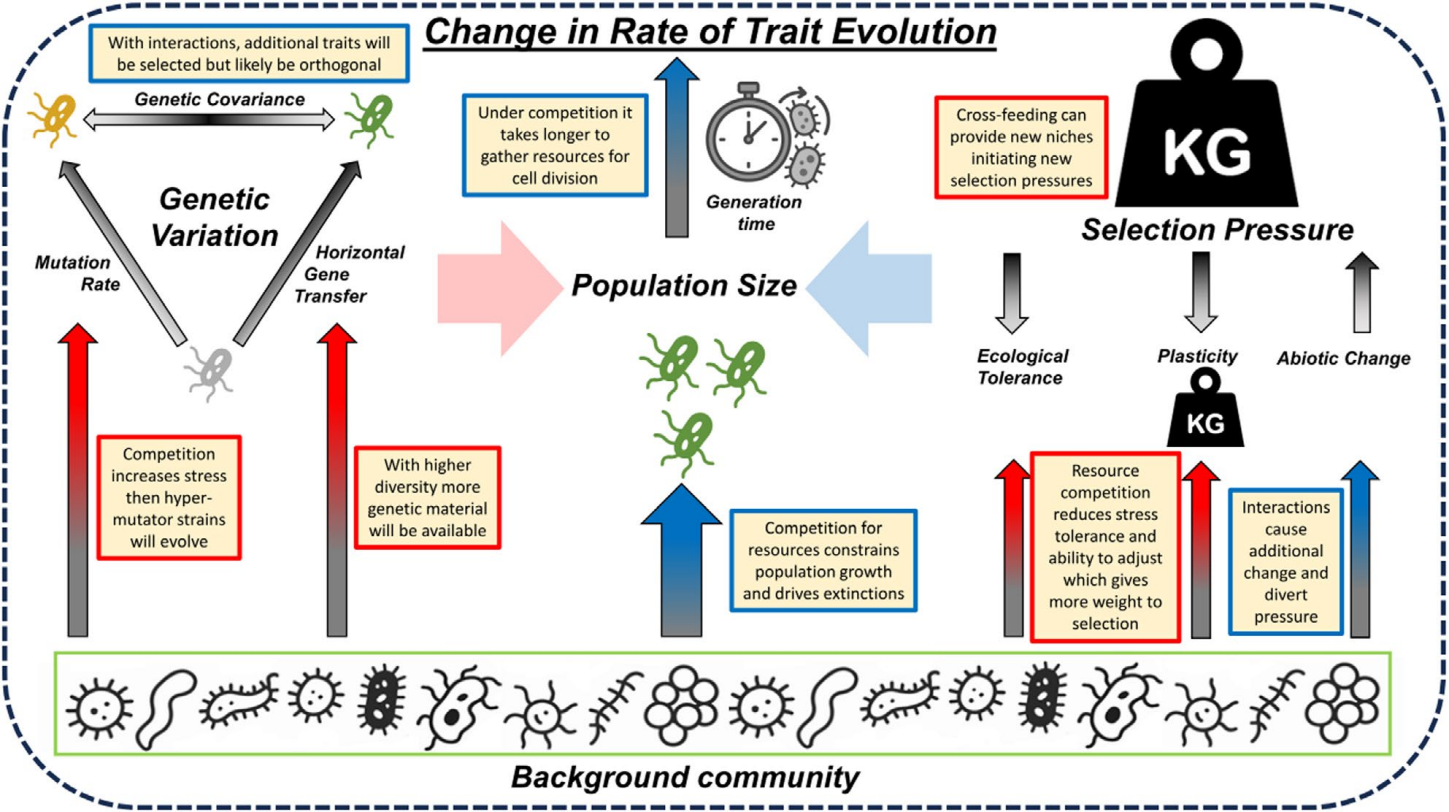
Species interactions can influence evolutionary rates in multiple ways. The rate of evolution of a species is set by the amount of genetic variation and the strength of selection, which are both canalised through population size and generation times. These factors are directly impacted by variables (e.g., mutation rate or ecological tolerance), which each can be either enhanced or suppressed by interspecific interactions. Conceptual ideas are given on how species interactions may modify evolutionary trajectories (yellow boxes, blue indicates a delaying and red an enhancing effect). For example, a background community (green box) may have a pronounced limiting effect on the population size of a species if there is high competition for resources (bold blue error in centre). However, the new adaptation emerged from increased horizontal gene transfer that was only possible as multiple species were present (red arrow, left side). This example may be comparably relevant, as horizontal gene transfer is assumed to be more likely between phylogenetically related species, but this may also increase the niche overlap. The relative importance of various effects is certainly highly case‐specific. In most cases, there are several scenarios that either constrain or facilitate evolution, and our summary is not exhaustive. Icons were generated using Microsoft Copilot.

To enhance our understanding of how evolution in bacterial communities can be directed, as outlined above, a detailed understanding of the underlying mechanisms will help. In principle, evolution increases with the number of generations, and its rate depends on the strength of selection and the presence of heritable additive genetic variance (Hendry 2016). Evolution proceeds at a faster rate when the selection gradient acting on phenotypes is steeper, and the population harbours more additive genetic variance (Schluter 2000). Genetic variance is influenced by population size, mutation rate and mutation effect size, as well as recombination rate and genetic covariances, while the strength of selection can be amended by ecological tolerance, phenotypic plasticity and the rate of environmental change (Barraclough 2019b), and all these aspects can be modified by interspecific interactions (Figure 3). Overarching, one of the most important factors associated with evolution is population size; competition will likely decrease population sizes, while cross‐feeding will increase them. Further, multiple biotic interactions may also impact generation times. If species, for example, can exploit new cross‐feeding products, this may allow faster cell division rates, whereas, under increased competition, cells may accumulate resources to persist in adverse conditions. There are, however, many more ways in which communities can impact the evolution of a focal species.

### 4.1 Natural Selection

The strength of selection can be modified by competitors when selection would pull a population towards exploiting a resource providing increased fitness, but a co‐evolving competitor effectively consumes this resource (decreasing adaptive peak), which results in successive pre‐emption of this ecological opportunity, thereby reducing selection (Osmond and de Mazancourt 2013). As such, the selection gradient β of an adaptive trait, the vector pointing to the nearest or steepest peak on the fitness landscape, may decrease if the peak is virtually depressed by competitors consuming the niche space, which would support the relevant fitness increase. Depending on the overlap of the fitness function between focal species and competitors, divergence may be promoted if there is limited interference or canalised if there is pronounced overlap (Roughgarden 1976; Slatkin 1980). Weak competition is predicted to increase selection and to emphasise divergence, but strong competition will constrain adaptive radiations by smoothing fitness surfaces (Slatkin 1980; Van Cleve and Weissman 2015). Further, selection is determined by the stress experienced; while, for example, low pH environments may have a limited effect on a population provided high‐quality resources are available, alleles that increase pH tolerance may experience little benefit. If, however, competitors remove these high‐quality resources, adaptive alleles for increased pH resistance may gain extra importance as physiological costs increase. Alternatively, cross‐feeding may provide more metabolisable resources and reduce stress, which may in turn rescue crucial genetic variation from extinction. Similarly, plasticity, the ability of an organism to express a different phenotype, may increase or decrease evolutionary rates (Chevin et al. 2010), and interactions may modify how organisms respond to changes. Again, if enough resources, and therefore energy, are available, plastic modifications to respond to stress should be more easily expressed (e.g., more ion pumps due to pH stress). If, however, competitive stress plays out, it seems plausible that critical resources are no longer allocated for plasticity changes. Finally, the selection pressure depends on the rate of environmental change, and a higher magnitude of change is likely to cause more selection. Biotic interactions are likely to add extra pressure to abiotic changes and thus increase the rate of environmental change but may also result in multiple orthogonal selection pressures.

### 4.2 Genetic Variation

**FIGURE 4:**
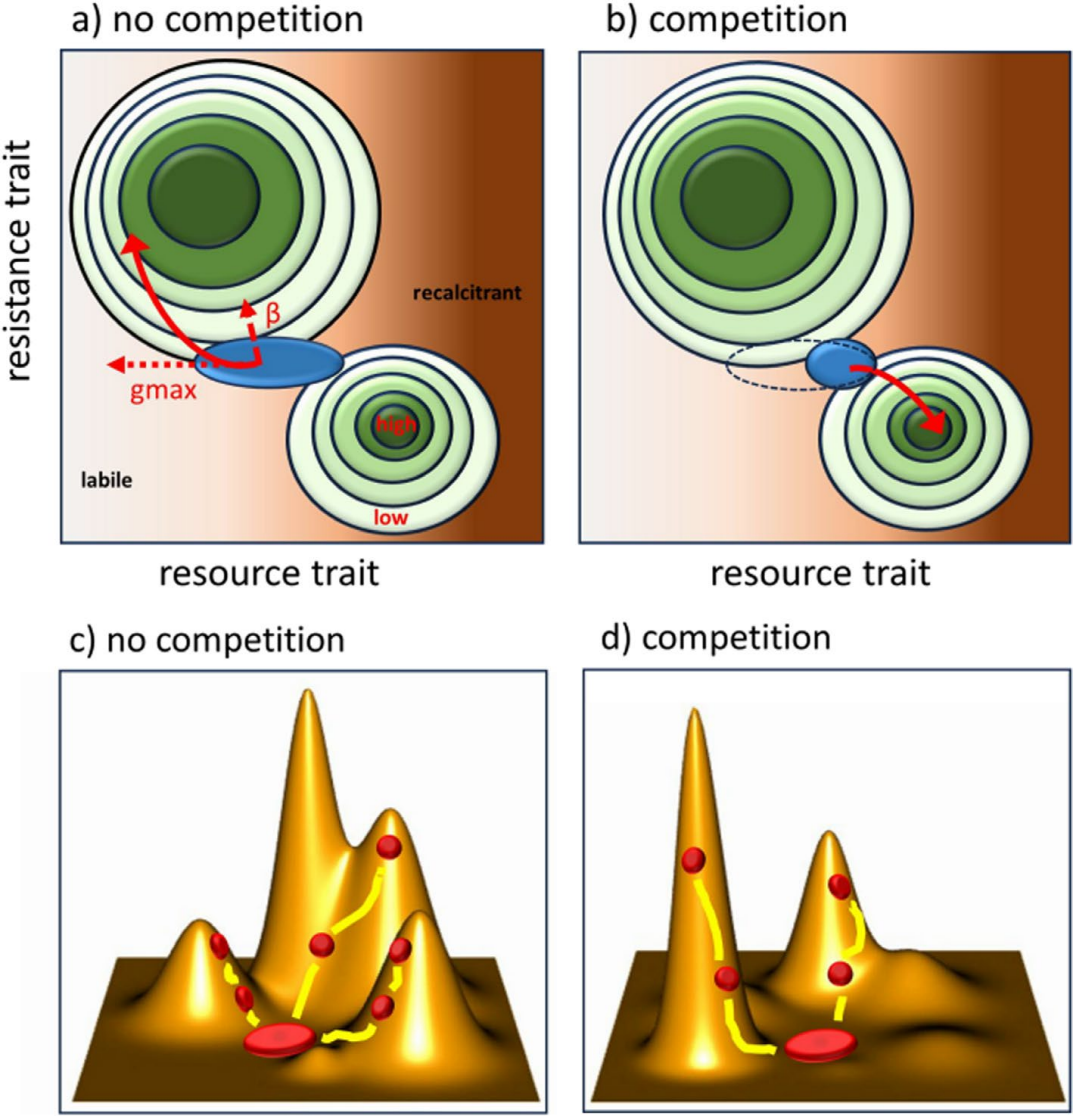
The adaptive pathway of a population on the fitness landscape can be biased. This hypothetical landscape consists of resources that range from labile to recalcitrant (darker brown background) and increasing levels of resistance. (a) An individual population with genetic variation (blue ellipse) finds itself on this landscape where both resource and resistance, traits display fitness peaks (green circles). The selection gradient β (red dashed arrow) pulls the population mean towards the steepest fitness increase (green circles with darker colours indicating higher fitness). Without competition, the population expresses extended variation and can exploit many different resources. The population initially evolves along the line of least resistance (*g*_max_, red dotted arrow) caused by the genetic covariance. The evolutionary pathway (red curved arrow) does not follow the selection gradient, but is biased by the greatest amount of genetic covariance. In this example, resistance evolution hinges on consuming more labile resources (compensating for the costs of resistance). The vector *g*_max_ does little counteract evolution and only slightly deviates the evolutionary trajectory from being pulled by the strongest selection gradient. Moreover, adaptive radiation and divergence into two sub‐populations is also possible if selection for a resource niche shift is equally strong as for resistance. (b) Under competition, ecological opportunities may be limited, and the population has to consume a narrower set of resources, which is a more recalcitrant side. As less of the genetic covariance can be expressed, *g*_max_ is limited, and the population may be more likely to evolve towards using recalcitrant resources, at the cost of resistance. Alternatively, cross‐feeding may turn the picture and provide more labile resources that facilitate the evolution of resistance. This thought experiment also raises the question if the evolution of an abiotic trait is resting on a shift in resources, or if abiotic adaptations mainly evolve independently. On top, it is well possible that fitness benefits of adaptive alleles change under biotic interactions and thus cause a modification in the slope of the fitness peaks. (c) Without competition, the fitness landscape for a population may display high ruggedness. With various niches available, a population (red) may diversify into different ecotypes (yellow lines). Potentially, the highest fitness peak is missed because the adapting population is trapped on a nearby local peak. (d) When other species consume resources, these are limited, and thus the fitness advantage decreases, which smoothens the landscape. However, it is less likely that the adapting population is trapped on a local peak and gets diverted from the original optimum. A common observation, however, is that new niches are created by metabolite excretion, which can form new adaptive peaks. These peaks are potentially rather steep and support large fitness increases.

Increased genetic variation in populations can enhance evolutionary rates to an extent that dominance and exclusion patterns are turned into competing bacterial communities (Scheuerl et al. 2019). In larger populations, the amount of standing genetic variation may be higher, but competition may reduce densities and, in the process, purge rare genetic variants that may be detrimental for future adaptations. Genetic variation is further directed by mutation inflow and mutation effect sizes; alleles may convey a potential small fitness advantage for recalcitrant products, but if more rewarding resources are present in the environment, these alleles may not unveil their capacity. Moreover, under stress, hyper‐mutator strains are more likely to evolve, which changes the inflow of new mutations (Barrick et al. 2009). In communities, genetic variation may increase through recombination during horizontal gene transfer via transformation, transduction and conjugation, which may increase if more genetic material (e.g., from cell lysis) is present. Moreover, even epistatic interactions between adaptive loci may be revised (Poelwijk et al. 2007). If two loci altering labile sugar metabolism (e.g., by transporter and enzyme changes) yield high fitness increases on their own, there may be little additive effect when recombined (de Visser and Elena 2007). When competition drives molecule concentrations lower at an increased pace, the individual loci may yield less advantage, but both loci together may provide the adaptive advantage, thereby reversing a negative epistatic interaction towards an additive pattern. Even the sign of mutations may revert, and modifications that are harmful under ecological versatility may provide benefits under more stressful environments. Moreover, adaptive traits may be correlated with the genetic covariance of another trait (*g*_max_), pointing in a different direction (Figure 4a). Correlation between these traits will bias *β* towards *g*_max_ (Schluter 2000). If, as in the hypothetical example, *g*_max_ is based on resource pattern, interaction between species will either condense (Figure 4b) or expand, the amount of genetic covariance, causing a shift in the bias.

Taken together, interactions between species potentially may deeply modify adaptive fitness landscapes. When particular trait combinations are yielding higher fitness than others, populations will be pulled towards adaptive peaks (Van Cleve and Weissman 2015), and with little competition, multiple peaks may be available and harbour opportunity for diversification, displaying rugged fitness landscapes (Figure 4c). Competition for resources is likely to dampen average fitness peaks and potentially smooth fitness landscapes. This is likely resulting in a canalisation towards the most promising trait combinations (Figure 4d). The interaction between species is also likely creating new niches, for example, by cross‐feeding, which may yield large fitness increases, pulling trait combinations in new directions.

In principle, all the factors affecting evolutionary rates can be impacted by interspecific interactions both in facilitating and constraining ways, and how interactions modify evolution is most likely very case‐specific. Supporting this, data suggest that the evolution of a focal bacterial species is well explained by interactions with the specific background community; whether a species evolves in a community depends very much on the specific biotic background and not only on more general factors, like biodiversity or genomic malleability (Scheuerl et al. 2020).

## 5 Future Research Directions

### 5.1 How Can ‘Ecological Opportunities’ Be Characterised in Greater Detail?

With interactions based on resources, the difficulty is that environments hold thousands of different carbon molecules that may serve as food for heterotrophic bacteria. Regrettably, only a tiny fraction of these molecules are described in detail, and metabolic pathways are characterised (Hu et al. 2022; Sheridan et al. 2022). Thus, large numbers of resources may potentially be available as niches that are currently widely unknown, without which, however, we are unable to accurately understand the α‐niche space in which these communities inhabit. Only if this vast number of resources can be described and how they are metabolised by bacteria will a true understanding of ecological niches and their role in microbial evolution be possible. With the breakdown of recalcitrant resources being less energy efficient, it can thus be speculated that species which are pushed to use them have limited capacity to evolve other traits. Following this, recalcitrant resources may foster more syntrophic interactions between species, which increases biodiversity. Alternatively, if specific key species that initiate the breakdown of recalcitrant molecules are missing, this may limit diversity (Gralka et al. 2020).

**FIGURE 5:**
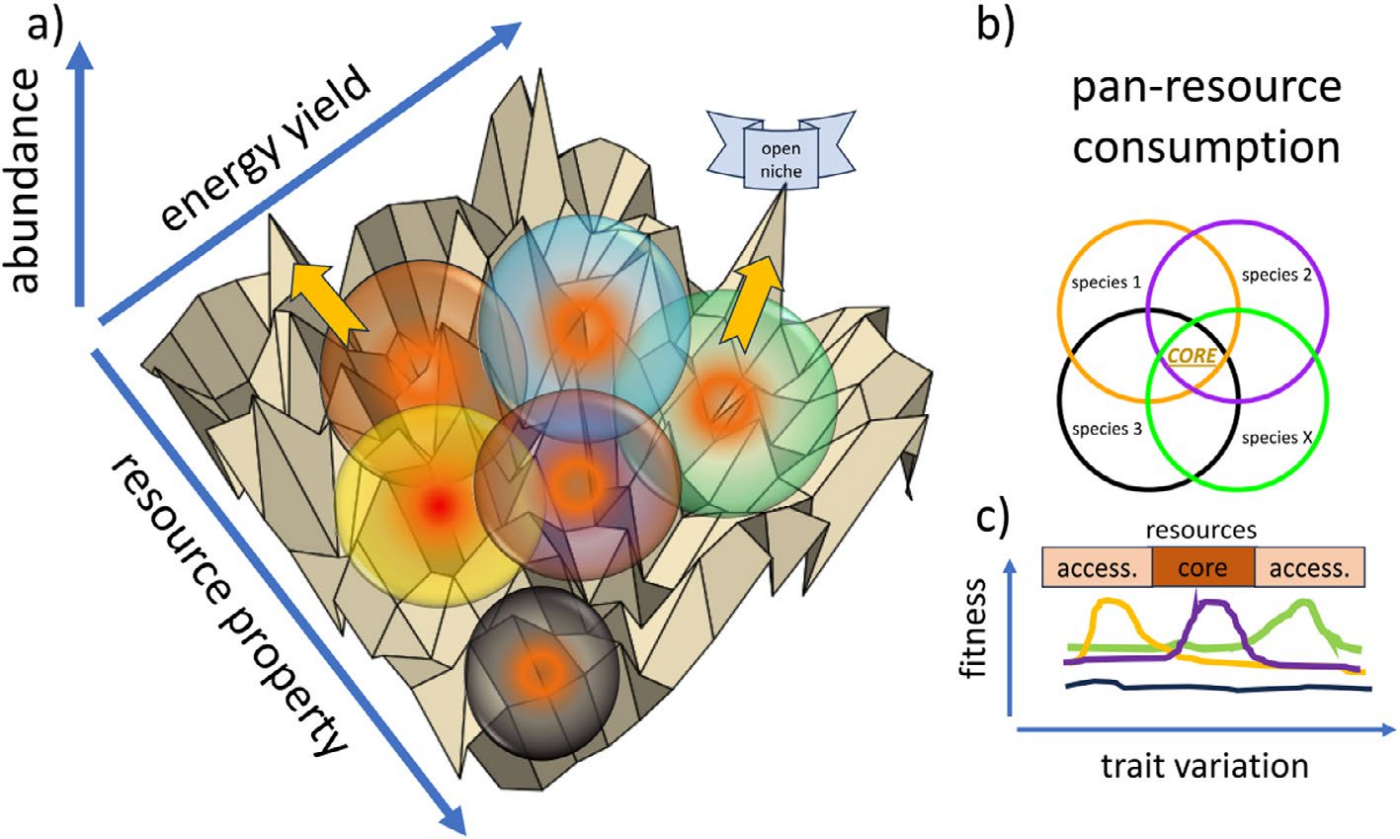
Exploring resource landscapes, how bacteria occupy niches and how they share them will yield important insight. (a) Characterising the present chemical diversity and classifying it into categories of metabolism should provide detailed insight into which α‐niches (and abundance of) are present in natural environments, how they are consumed, and which by‐products are released that can be used for cross‐feeding. This would also illuminate how niches of species overlap and which resources are underexplored, serving as potential open ecological niches. In the given example, there is considerable niche packing. The black species evolved using a resource of extra property with low energy yield, but escapes competition. The green and the blue species may both have access to an open and rewarding α‐niche, but green may be more likely to evolve (yellow arrow) as existing metabolic traits are more suitable, and this will reduce competition with the purple species. (b) Like pan‐genome plots, pan‐resource plots could unveil what core resources these species of the community use (shown for a subset of species for simplicity), and which are accessory or alternative resources that are used only by very few species. In many environments, this core‐resource fraction may be rather high; thus, it will be clear which alternative resources are underexplored and may support new adaptation. (c) Overall, selection gradients for different resource niches are likely to be very different for each species. The purple species may have little opportunity to exploit accessory resources (resources that are not used by many other species), and selection may act in a condensing manner, so the population evolves to dominate the core resources and becomes a dominant competitor. The orange and green species both may evolve using accessory resources and, by doing so, escape strong competition. The black species may not evolve but remain in its niche (additional species are omitted for simplicity). In summary, of key interest will be whether traits exploiting these resources can evolve from existing traits, or if new key functions have to be evolved to seize them. But our data are far too limited to draw precise conclusions.

Bacterial modification of the niche landscape has also been shown to determine how species behave in a community, with secondary metabolites used and produced in unequal measures (Morgan et al. 2020). Thus, how diversity and identity of resources impact biotic interactions and, subsequently, community assembly is an important open question (Dal Bello et al. 2021; Estrela, Sanchez‐Gorostiaga, et al. 2021; Pacheco et al. 2021). Recent years have seen exciting new developments in describing the present resource molecules in environments; ultra‐high resolution mass spectrometry is unveiling molecular formulae and has the power to elucidate these details (Kellerman et al. 2014). The first results are very promising, suggesting that these methods can give insight into microbial resource niches in unprecedented detail (Sheridan et al. 2022). Ongoing works try to characterise this molecular diversity before and after bacterial activity, and this will provide insight into which resources are used, how they are partitioned and how they are metabolised. These initial data suggest that environments can hold several thousand different chemical formulae, and bacterial activity even increases this number, presumably by breaking larger molecules into smaller fractions of variable size (Scheuerl, personal communication; Sandor 2025). Thus, many new niches could be created even from a few high‐molecular‐weight molecules (Fonvielle et al. 2025). In nature, this results in a vast diversity of chemical formulae (Kellerman et al. 2014; Tanentzap et al. 2019), and bacteria may have evolved traits to exploit all of them. With such data, fine‐scale resource landscapes could be created that illuminate which resources are present, their abundance and how efficiently metabolised they are by different bacteria (Figure 5a). Tools are now established to estimate bioavailability, which could be used to categorise resources into classes (D'Andrilli et al. 2015). Thus, resources could be categorised into formulae, abundance, bioavailability and energy yield and consumption or production could be tracked. This way, a precise picture of the available resources, and thus the present α‐niches, of complex environments can be created. Of course, this landscape will be malleable, as bacterial activity may continuously consume and produce molecules. For many environments and the bacterial communities that inhabit them, the core resources that most present species compete for may be rather high; thus, characterising the accessory resource niches should yield important insight into how different bacteria partition resources and coexist in nature (Figure 5b). This would also allow predictions of which resource niches different bacterial species will likely evolve to consume. In this way, selection surfaces for species may be rather different. For some species, selection may act to focus even more on core resources that are highly rewarding but competition is high, while other species may evolve to consume accessory resources that are underutilised (Figure 5c). Of special interest will be whether the ability to exploit these resources can evolve from existing traits (modification of existing related pathways) or if new key functions have to be evolved to seize them. Such resource landscapes would give valuable insight into which niches are available in a species' surroundings, mirroring fitness landscapes and allow prediction of which species may diversify into alternative niches.

BOX 2 **Open Questions to Be Addressed.**

There are a number of outstanding questions that need to be addressed to fully appreciate how these two ecological and evolutionary processes combine.

When we think about resources as ecological opportunities:

- Which resources (core vs. accessory resources) do different species use, and how do species within communities partition them?
- What can bacteria metabolise on their own, and for what kind of resources are collaborative networks required?
- Are there resources that foster collaborative behaviour or are there some that lead to enhanced competition?
- Can β‐niche traits evolve without α‐niche trait shifts?
- Are α‐niche traits shifting due to standing genetic variation or due to key innovations from new mutations?
- Do different resources cause different evolutionary changes of β‐niche traits?

When we think about biotic interactions:

- In what conditions will species interactions amend evolutionary trajectories or not?
- Are higher‐order biotic interactions important, and what is the best way to measure them? Alternatively, are the main drivers pairwise interactions as envisioned previously?
- Do strong interactions have a pronounced effect compared to widespread weak or diffuse interactions among many species?
- Do facilitative interactions, where species rely on partners, increase or decrease the amount of evolution in a community context?
- Are negative (e.g., competitive) or positive (e.g., mutualistic) interactions on average more important?

### 5.2 Can Our Understanding of How Species Evolve in Nature Be Improved?

So far, our knowledge about community evolution is still in its infancy; more data are needed (Barraclough 2019b), and a wide range of open questions remain (Box 2). Not only is there a paucity of studies investigating evolution in a community context, but we need more studies that explore how interactions themselves evolve (Piccardi et al. 2019). Thus, how stable are they over evolutionary timescales? Laboratory‐based studies found evolution of neutral interactions starting from competitive situations (Evans et al. 2020; Fiegna, Moreno‐Letelier, et al. 2015; Fiegna, Scheuerl, et al. 2015; Lawrence et al. 2012); but then why are these competitive at all if species coexist in nature? Interaction strengths and signs are ephemeral in different habitats and at different times, making their measurement difficult and maybe even calling for new concepts. Two species may compete for resources in one environment but facilitate each other in a slightly modified environmental context (Beilsmith et al. 2020), which raises the question of how transient interactions impact population dynamics. Direct interactions, like excretion of toxic metabolites, are maybe easier to study, as there are often quantifiable molecules that are used to kill competitors, but in low‐nutrient environments, as often found in nature, it is not clear how relevant this is (Lawrence and Barraclough 2016). Further, a clearer picture is needed of how important population sizes are, if generalists or specialists are more evolvable and how higher‐order interactions integrate. Moreover, co‐evolved networks may show greater stability against disruptive effects imposed by an invader (Rivett et al. 2018). Directly interacting species in the co‐evolved community may be potentially protected if better integrated into the overall network. The question about stability in co‐evolved communities still needs to be much better explored, as evolution may either stabilise or destabilise communities (Loeuille 2010), which implies the question of whether eco‐evolutionary feedbacks are important in complex communities.

Many of these questions hinge on the issue of how to design experiments so component species and community members can be tracked and eventually recaptured. This is a difficult question, particularly in bacteria, and probably requires the development of more tools. Species may be marked by cytosolic dyes, genetically tagged and carry antibiotic resistance genes, allowing them to be re‐isolated, but all these tools can alter cellular processes and thus often come with fitness costs that render the carrying organism unfit in the current community or environment. The most exploited tool is using sequencing technologies; however, exclusively relying on molecular data (e.g., metagenomics) will deprecate important information gained from phenotypic assays, particularly, this phenotypic information is so valuable for us. After all, it is the phenotype, not the genotype, which is the relevant unit to understand and predict processes that affect ecosystem function (Hendry 2016). While we certainly can gain highly valuable information from omics tools, holding the phenotype in hand is commonly the key to deep understanding. One study used dialysis bags, allowing tracking and re‐isolation of focal species after evolutionary time (Scheuerl et al. 2020). This is a mouldable approach, but physical cell–cell contact is precluded, which may be important in some situations.

**FIGURE 6:**
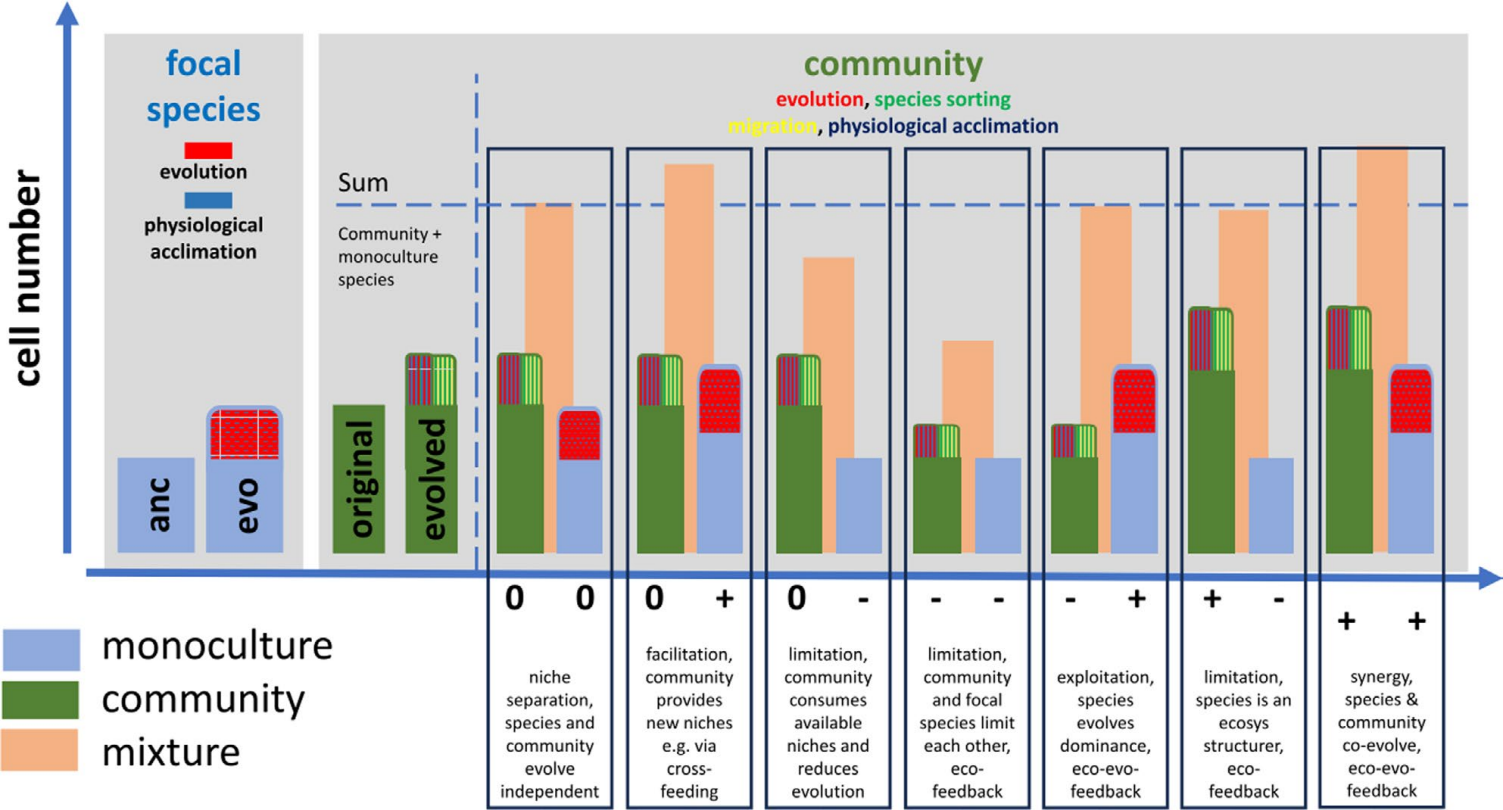
Experimental design to study how bacterial species interact with complex communities. To explore how individual species and complex communities co‐evolve, detailed experiments will be needed. Under selection, populations will undergo physiological acclimation and accumulate heritable phenotypic variation, which can be estimated by comparison with the ancestor. In parallel, diverse communities will change in composition and function over evolutionary timescales by ecological sorting (frequency changes of species), migration (loss or gain of species) and component species will acclimate as well as evolve. Previous works suggested comparing traits of different species in isolated cases and predicting from these cases where species over‐ or underperform in mixture, to estimate interactions (Foster and Bell 2012; Mitri and Foster 2013). To estimate how communities modify species evolution and maybe also how this loops back from the species to the community, similar approaches could be taken. When considering together, comparing evolved species, their ancestor and the co‐evolved community, the performance (e.g., cell counts) of each isolated component could be measured and compared to the performance when species and community grow together. A prediction from the isolated case could be made and compared to the observed performance. As an example, a species is re‐isolated from the community or from monoculture after selection and its evolutionary change is evaluated by comparison with the ancestor. Similarly, the starting and final communities could be compared. Now, evolved species and ‘evolved’ communities are grown together. When the observed measurement meets the prediction, the species evolved independently from the community and found its own niche. If it is less than the prediction, and it is the evolved species that is growing less, then the community constrained evolution. If it grows more, the community facilitated evolution. In parallel, the effects of the species on the background community could also be estimated.

For monitoring and assessing interactions, first between species but also between focal species and communities, research will benefit from new concepts. Previous approaches to studying bacterial interactions suggested growing bacterial species first in isolation and then in mixture (Foster and Bell 2012). From the isolated case, predictions can be made about how the mixture should grow if there is no interaction between the species, with over‐ or underperformance suggesting interactions (Fiegna, Moreno‐Letelier, et al. 2015; Mitri and Foster 2013). A similar approach could be used to study how co‐evolved communities affect component species evolution (Figure 6). Once calibrated with ancestral performance, comparing mixed performance with predicted performances would probably allow estimation of how communities direct species evolution. In such a design, focal species may genetically evolve new phenotypes but also respond by phenotypic plasticity and physiological acclimatisation, which can be disentangled by comparison with the ancestor (Bennett et al. 1990; Scheuerl et al. 2020). Potentially, not only could evolution between the evolved and ancestor be determined, but also how both are affected by the community.

In parallel, communities should also be tracked, not only to explore how they affect focal species, but also to see if there is feedback and how focal species affect entire communities. Over evolutionary timescales, communities may change by ecological sorting (frequency changes), dispersal (immigration and extinction), physiological acclimatisation of members, as well as evolution of species, but individual species may have the capacity to modify each component. A combination of omics tools and functional and phenotypic assays (e.g., Goldford et al. 2018; Rivett and Bell 2018; Scheuerl et al. 2020) could be used to illuminate the underlying processes within these communities, and several predictions could be tested. For example, when maintained in a single‐carbon environment, for example, glucose, a distinct, sugar‐loving community should emerge, but some biodiversity is maintained, which is fuelled by cross‐feeding of metabolites (Goldford et al. 2018). This cross‐feeding may mainly evolve in rare species as this new niche emerges. When the number of supplied resources increases, more species can coexist and diversity increases with predicted diversity of metabolites (Dal Bello et al. 2021; Pacheco et al. 2021). Not all the supplied resources can be used by all the different bacteria, and not all resources yield the same energy; thus, adaptations to streamline metabolic pathways consuming these sources at the cost of other resources are likely. In complex environments, multiple species are observed to partition their functionality based on abundance (Rivett and Bell 2018). Potentially due to these mechanisms, communities assemble even in complex environments in similar ways; however, small differences in initial composition can tilt towards different outcomes (Pascual‐García et al. 2025). Even in natural settings, where composition and function are influenced by dispersal (Rivett et al. 2021), there are still distinct patterns that emerge within the microbiomes (Shabarova et al. 2021), but how evolution impacts this is unknown. Research has shown that predictability is comparably high under controlled conditions (Estrela, Vila, et al. 2021; Goldford et al. 2018; Pascual‐García et al. 2025), so detectable changes can probably be attributed to evolutionary or ecological processes. Thus, replicated tests could unveil when communities converge or what determinants drive divergence and how ecological and evolutionary rules play out together. Experiments, as suggested above, would also allow exploring how individual species act on the community in return and offer ways to estimate if there are eco‐evo‐feedback loops (Hendry 2016; Schoener 2011), which would provide detailed knowledge of which mechanisms drive community fate in the long term. Maybe over evolutionary timescales, mainly ecological processes occur, but most of our data suggest that even in complex communities, evolution plays an important role and thus has huge potential to modify community composition and function. So far, ecological forces shaping bacterial communities have received quite some attention, whereas the evolutionary component is usually not well explored. Arguably, in many laboratory‐controlled systems where experiments lasted only a few days, this reasoning may be justified, but in reality, little data exist (Bennett et al. 1990; Chase et al. 2021; Rainey and Travisano 1998; Wheatley et al. 2021). Regardless, in nature, bacterial communities do not coexist for just a few days (generations), but at much longer timescales; thus, the role of evolution is important and should be investigated further. As highlighted above, co‐evolving bacterial communities can quickly expand a niche range (Adamowicz et al. 2020; Fiegna, Scheuerl, et al. 2015; Lawrence et al. 2012); they evolutionarily occupy vacant niches and consume resources more broadly in just a few days, therefore altering ecological dynamics (Martiny et al. 2023). Long‐term observations have provided the first evidence of rapid ongoing evolution in natural communities, emphasising the relevance of community ecology to evolution and ecosystem function (Bendall et al. 2016; Rohwer et al. 2025). Again, how changing interactions, based on α‐niches, finally correlate with the evolution of β‐niches needs careful consideration.

## 6 Solving the Uneasy Alliance of Ecological and Evolutionary Research

Blocking blooms of specific bacteria ecologically by adding bacterial communities is a straightforward idea (Spragge et al. 2023), and developing probiotic food supplements building on this is a quickly expanding market. But a recent review concluded that probiotics are not yet effective enough to hit this goal (Rueda‐Robles et al. 2022). This is just one example of how bioaugmentation requires the complete understanding of the interplay between ecological and evolutionary dynamics (Liu and Suflita 1993). Including the bipartite nature of α‐niche trait and β‐niche trait adaptation into evolutionary microbiome research should mitigate continued ecological shortcomings in this field, even if uneasy scepticism is difficult to address. Applying the ecological drivers of community change to evolutionary trajectories should be exploited to modify the evolution of focal species after knowing how interactive networks permit the broadest consumption of ecological opportunities. As such, after characterising the niche occupation of focal species and the resources they will evolve to exploit, ecologically similar species could be pre‐evolved to pre‐empt relevant α‐niche space. Additionally, specific resources could be supplemented to enhance adaptive radiations of key‐competitor species, but not focal species, so that these competitors find an opportunity to evolve ecological dominance. This way, focal species could be forced to evolve along resource niches that do not support ameliorating the costs of other adaptations.

Of course, this framework is rooted in theoretical concepts and laboratory testing. To increase the impact on real‐world problems, whether these be host‐associated or environmental, empirical data need to be generated. Such tests should also consider the potential impact of higher‐order interactions and stochasticity during the assembly process, which we have not considered here. Further, there are additional aspects contributing to the evolutionary process of bacteria in nature, like the impact of mobile genetic elements (e.g., plasmid conjugation) or spatial constraints (biofilms) that lead to aggregations of populations in specific places. Also, top‐down processes across trophic levels, like predation by ciliates or bacteriophages, are likely to amend evolutionary trajectories, which we have only briefly considered here, and emphasise the great potential natural complexity harbours. We welcome research into these interactions, which would increase the applicability of our framework.

## 7 Concluding Remarks

In summary, we present a conceptual framework by which species' evolution of β‐niche traits is impacted by community interactions when α‐niche competition is involved. With this concept in mind, approaches to seize the eco‐evolutionary potential of communities to direct adaptation into specific directions can be envisioned, bridging the divide between ecological and evolutionary research and propelling our ability to directly manipulate evolution in natural microbiomes.

## Author Contributions

**Thomas Scheuerl:** conceptualization, writing – original draft, visualization, writing – review and editing, project administration, funding acquisition. **Damian W. Rivett:** visualization, writing – review and editing.

## Funding

This was supported by an early‐career grant from University of Innsbruck (316807), Tiroler Wissenschaftsförderung (325779), Marie Skłodowska‐Curie Actions (MSCA) Postdoctoral Fellowship (101067338) and Engineering and Physical Sciences Research Council (EP/X024830/1). DWR was supported by Vertex Pharmaceuticals Limited Research Investigator Award (IS‐S‐202‐1‐109666).

## Conflicts of Interest

The authors declare no conflicts of interest.

## Data Availability

Data sharing is not applicable to this article as no datasets were generated or analysed during the current study.
